# New Insights into the Combined Antiviral Effect of Extracts from *Nerium oleander* and *Boswellia sacra* Against Respiratory Syncytial Virus: A Preliminary Report

**DOI:** 10.3390/pathogens15030260

**Published:** 2026-03-01

**Authors:** Rebecca Piras, Luca Virdis, Valeria Manca, Marta Cogoni, Vanessa Palmas, Matthew G. Donadu, Aldo Manzin, Giuseppina Sanna, Luay Rashan

**Affiliations:** 1 Department of Biomedical Sciences, Section of Microbiology and Virology, University of Cagliari, 09042 Monserrato, CA, Italy; 2 Hospital Pharmacy, Giovanni Paolo II Hospital, ASL Gallura, 07026 Olbia, OT, Italy; 3 Research Center, Dhofar University, Salalah 211, Oman

**Keywords:** natural compounds, *Boswellia sacra*, *Nerium oleander*, viral diseases, extract mixture, respiratory syncytial virus, broad-spectrum viruses

## Abstract

In recent years, the emergence of drug-resistant pathogens and the limitations of current therapies have highlighted the need for innovative strategies to combat emerging viral infections. Natural compounds, derived from plants, are playing an increasingly significant role in the research of novel and effective therapies. *Boswellia sacra*, a frankincense-producing tree widely distributed in Yemen and Oman, and *Nerium oleander*, a common ornamental and medicinal plant, are examples of plants with well-documented antimicrobial properties. Their extracts have demonstrated good activity against a wide range of infections, which is attributed to the anti-inflammatory and immunomodulatory compounds they contain. Based on these findings, we assessed, in vitro, the broad-spectrum antiviral activity of combined extracts obtained from *Boswellia sacra* and *Nerium oleander*. The extract mixture NOBS7(1) was found to be active against the respiratory virus RSV, Herpesvirus simplex type 1, and Coxsackievirus 5. Furthermore, a combination of cell-based assays was performed to provide additional insights into their potential mechanism of action.

## 1. Introduction

The search for natural compounds with antiviral properties has gained significant attention in recent years, particularly in the context of emerging viral infections and resistance to conventional treatments. They serve not only as direct treatments but also as structural templates for designing potent, broad-spectrum, and sustainable antiviral drugs. Among the studied phytocompounds, *Boswellia sp*. and *Nerium oleander* stand out for their broad spectrum of biological activities, including immunomodulation [1], anti-inflammatory and antimicrobial activity [2], and potential antiviral effects [3]. *Boswellia* species extracts have been used in traditional medicine for many years to treat inflammation and infections. The main bioactive components of these extracts, like boswellic acids and terpenoids, with their derivatives, have demonstrated anti-inflammatory [4], antitumoral [5], immunomodulatory, and antiviral properties, suggesting a potential role in various viral diseases [3,4,5,6]. Boswellia species include *Boswellia sacra*, which is an economically and ecologically important frankincense-producing tree [7] with analgesic, anti-inflammatory, antitumoral, and antiviral properties, which are attributed to the presence of several bioactive components and derivatives in the lipidic fraction of its extracts [8]. Boswellic acid (BA) mixtures derived from frankincense exhibit potent antiviral properties, specifically against Herpes Simplex Virus type I (HSV-1). Mechanistically, BAs and *B. serrata* extracts act as host-directed antivirals on HSV-1 replication by blocking the NF−κB signaling pathway, a critical component for viral proliferation. Similarly, *Nerium oleander*, an ornamental and medicinal plant, contains cardioactive glycosides and phenolic compounds with cytotoxic and antiviral properties [9]. Standardized and well-dosed extracts of this plant might interfere with the replication of specific viruses [10]. The cardiac glycoside, oleandrin, has been reported to have antiviral activity specifically against ‘enveloped’ viruses, including HIV, HTLV-1 [11], and more recently, SARS-CoV-2 [12]. Our research group previously reported the anti-poliovirus activity of *Nerium oleander* aqueous extract [10]. 

This work aims to enhance the in vitro potential of *Boswellia sacra* and *Nerium oleander* as antiviral agents, and to examine the antiviral effect when their extracts are prepared in combination. The antiviral activities of *Boswellia sacra* extracts alone are reported in Table S1, Supplementary file, while the antiviral activities of *N. oleander* extracts are described by us [10] and other authors [11].

## 2. Materials and Methods

### 2.1. Plant Material

The date of the previous collection was November 2001, when weather conditions were cold (wintertime in Jordan), whereas the date of collection for the current study was July–August 2019, and the weather conditions were mild (summertime in Jordan). The specimens were deposited at the Royal Botanic Garden and Jordan University (Amman, Jordan). The voucher codes are SN/NC1 and SN/NC2. 

*Nerium oleander (Apocynaceae)* leaves were collected from Jordan and were taxonomically identified by direct comparison with authenticated samples of the herbarium of the Biology Department, College of Science, Jordan University, Jordan.

Oleo gum resins were collected from verified *Boswellia sacra* Fluck trees of Wadi Doka (Najdi type resin) in the plateau region north of Salalah, Sultanate of Oman, during 2023. The sample was collected by the traditional method. This region experiences a desert climate, with low rainfall (<100 mm annually) and sharp temperature variations throughout the day.

### 2.2. Extraction Methods and Preparation of the Combined Extract

#### 2.2.1. Extraction of Nerium Oleander Oil

Breastin is a standardized cold-water extract obtained from the leaves of *Nerium oleander*. The preparation of the extract has been previously described [13].

Sterile, freshly ground leaves (200 g) of *N. oleander* were soaked in distilled water (1000 mL) for at least 8 h at room temperature. After filtration, the volume was adjusted to 350 mL to yield a clear dark brown extract, which was then lyophilized according to the previously described method [13].

#### 2.2.2. Extraction of Boswellia Sacra Gum Resin (BSR) Acid Fraction

The frankincense oleo-gum resin was stored in a freezer for more than 12 h to reduce its natural stickiness. The frozen resin was subsequently ground in a mortar and further processed using an electrical grinder for approximately 2 h to obtain a fine powder. A total of 200 g of the powdered material was transferred into a 5 L round-bottom flask, followed by the addition of 1 L of distilled water. Hydro distillation was carried out at atmospheric pressure using a Clevenger-type apparatus, and the essential oil (14.2 mL) was collected after 6–8 h of distillation. After hydrodistillation, the remaining mixture was allowed to cool for 4–6 h, forming the acid fraction. This fraction was separated into two layers: a dark-colored upper aqueous layer and a lower precipitate layer. The aqueous layer was lyophilized to yield 5.3 g of material, which was coded as BS8. The precipitate layer was washed with hot water and filtered using Whatman filter papers (grades 1, 2, and 3), then washed repeatedly with hot water (3–4 times). The filtrate was cooled to 0 °C, yielding an off-white precipitate. After 60 min, the precipitate was washed several times with cold distilled water, dried under vacuum, and ground using an electric grinder to obtain approximately 80.0 g of powder, coded as BS7. For preparation of the combined extracts NOBS7(1) and NOBS8, respectively, 20 g each of BS7 and BS8 were finely ground (particle size 3–5 mm) and separately mixed with 25 mL of standardized Nerium oleander cold extract. The mixtures were stirred using a magnetic stirrer for 24 h at room temperature and subsequently filtered. The extract containing BS7 was immediately lyophilized and designated NOBS7(1). The BS8–N. oleander mixture was filtered and divided into two aliquots: the first was directly lyophilized and labeled NOBS8(2), while the second was boiled for 2 h prior to lyophilization and labeled NOBS8(3).

### 2.3. Chemical Profiling of Standardized and Lyophilized Extracts

The following chromatographic techniques were used for the chemical profiling and characterization of the extracts used in the present study, including extracts before and after combination: ultra-performance liquid chromatography–Orbitrap mass spectrometry[(UPLC–Orbitrap-MS) analysis was performed using an Orbitrap Exploris 120 mass spectrometer coupled to the Vanquish Flex UPLC system (Thermo Fisher Scientific, San Jose, CA, USA) at the Biopolymer Research Center for Advanced Materials (Sejong University, Seoul, Republic of Korea)])-based metabolomics approach for the annotation and analysis of various metabolites in these extracts; data comparison with tandem mass spectrometry (MS/MS); high-performance liquid chromatography–tandem mass spectrometry (HPLC/MS/MS); gas chromatography–mass spectroscopy. [Tandem mass spectrometry (MS/MS) analysis was performed, including triple quadrupoles (QqQ), quadrupole time-of-flight (Q-TOF), and ion trap systems (Thermo Fisher Scientific, San Jose, CA, USA) at the Biopolymer Research Center for Advanced Materials (Sejong University, Seoul, Republic of Korea)]. These techniques were previously described [13].

### 2.4. Cells and Viruses

Cell lines were purchased from the American Type Culture Collection (ATCC).

Cell lines supporting the multiplication of RNA and DNA viruses were Monkey kidney (Vero-76) [ATCC CRL 1587 Cercopithecus Aethiops], while cytotoxicity was also investigated in adenocarcinomic human alveolar basal epithelial cells, A549 cells [ATCC CCL-185], human colorectal adenocarcinoma, and Caco-2 cells [ATCC HTB-37]. The absence of mycoplasma contamination was checked periodically by the Hoechst staining method. Viruses were purchased from the American Type Culture Collection (ATCC). A virus representative of positive-sense, single-stranded RNAs (ssRNA+) was *Picornaviridae*: human enterovirus A71, strain BrCr (ATCC VR-1775), coxsackie type B5 (CVB5), and strain Faulkner (ATCC VR-185). The virus representative of negative-sense, single-stranded RNAs (ssRNA-) was *Pneumoviridae:* human respiratory syncytial virus (RSV) strain A2 (ATCC VR-1540); *Paramixoviridae:* measles virus (MV) Edmonston strain (ATCC VR-24); *Rhabdoviridae*: vesicular stomatitis virus (VSV) [lab strain Indiana] (ATCC VR 1540). DNA virus representatives were *Poxviridae*: vaccinia virus (VV) [vaccine strain Elstree-Lister] (ATCC VR-1549), Herpesviridae: human herpes 1 (HSV-1) [strain 17+]. Viruses were maintained in our laboratory and propagated in appropriate cell lines. The viruses were stored in small aliquots at −80 °C until use.

### 2.5. Cytotoxicity Assays

Vero-76, A549, and Caco-2 cells were seeded in 96-well plates at an initial density of 5 × 10^5^ cells/mL (1 × 10^5^ cells/mL for Caco-2), in Dulbecco’s Modified Eagle Medium (D-MEM) with L-glutamine and 25 mg/L kanamycin, supplemented with 10% FBS. Cell cultures were then incubated at 37 °C in a humidified 5% CO_2_ atmosphere in the absence or presence of serial dilutions of test compounds. The test medium used for the cytotoxic assay, as well as for the antiviral assay, contained 1% of the appropriate serum. Cell viability was determined after 72–96 h at 37 °C by the MTT method [14].

### 2.6. Antiviral Assay

All experimental assays involving viruses were carried out in a Biosafety Level 2 (BSL-2) laboratory (Microbiology and Virology Unit, Cittadella Universitaria di Monserrato). Compound’s activity against EVA71, measles, and RSV was based on the inhibition of virus-induced cytopathogenicity in Vero-76 cells acutely infected with an m.o.i. of 0.01 [15]. The compound’s activity against VV was determined by plaque reduction assays in infected cell monolayers, as described previously [16].

### 2.7. Cell Pretreatment Assay

A pre-attachment assay was performed by incubating cell monolayers with increasing concentrations of NOBS7(1) and NOBS8 or dextran sulfate (as a positive control) for 1 h at 4 °C, in order to allow interaction with the cell surface. After removal of unbound compounds, cells were further incubated with RSV for 2 h at room temperature and then washed and shifted to 37 °C. Plates were stained with Crystal violet, and the inhibition of virus-induced CPE was recorded using TECAN Infinite 200 Microplate Reader and an inverted light microscope, after 5 days.

### 2.8. Adsorption Assay 

Vero-76 cells grown in a 24-well plate were infected with RSV, with an m.o.i. of 0.1, in the presence or absence of fixed concentrations of compounds NOBS7(1) and NOBS8(3) (5 and 10 µg/mL, respectively). Multiwell plates were incubated for 60 min at 4 °C. The medium containing unadsorbed virus was then removed, and cells were washed twice with PBS and overlayed with the medium. After 5 days of incubation, monolayers were stained with Crystal violet, and the inhibition of virus-induced CPE was recorded using a TECAN Infinite 200 Microplate Reader and an inverted light microscope.

### 2.9. Virucidal Activity Assay

NOBS7(1) (1 µg/mL) and NOBS 8(3) (10 µg/mL) were incubated with 1 × 10^5^ TCID_50_/mL of RSV at either 4 or 37 °C for 2 h. The mixture without the test samples was used as the control. At the end of the incubation period, samples were serially diluted in media, and titers were determined on Vero-76 cells at high dilutions, at which the compound was not active. Virus titers were determined by endpoint dilution methods in Vero-76 cells.

### 2.10. Statistical Analysis

Cell-based experiments were independently repeated at least three times. The data are reported as mean ± standard deviation (SD). If not indicated, variation among samples was less than 15%. The statistical significance was calculated with an ordinary one-way ANOVA performed in GraphPad Prism (San Diego, CA, USA), * *p* < 0.05, ** *p* < 0.01, *** *p* < 0.001.

## 3. Results

### 3.1. Composition of Combined Extracts

As shown in Table S2, the chemical composition of the combined NOBS7(1) extract revealed a variety of compounds, including steroidal and non-steroidal glycosides such as digitoxigenin, neriifolin, and odoroside, as well as terpenoids, including monoterpenes, sesquiterpenes, diterpenes, and pentacyclic triterpenes, and coumarins and their derivatives.

NOBS8, on the other hand, was mainly characterized (Table S2) by fatty acid amides and triterpenoid constituents. The predominant compound was (Z)-9-octadecenamide (23.27%), and 24-norurs-3,12-diene-11-one (9.99%), viscinolide (9.40%), and β-amyrin (5.92%) were also detected as major components. According to literature data, the primary bioactive constituents of *Boswellia sacra* (frankincense) are boswellic acids (BAs). This group of pentacyclic triterpenes is found in the lipophilic portion (55–66%) of the gum resin [17]. Key compounds include β-boswellic acid, 11-keto-β-boswellic acid (KBA), and 3-O-acetyl-11-keto-β-boswellic acid (AKBA). The resin also contains an essential oil fraction of 5–15%, which is rich in monoterpenes and diterpenes [17]. Cardiac glycosides, glycones, and aglycones are the most significant bioactive constituents in *Nerium oleander* extracts. The primary cardenolide is oleandrin (the glycoside), which consists of a sugar moiety (glycone) and a steroid-like core (aglycone or genin), specifically oleandrigenin [18]. Other glycosides include neriin, digitoxigenin, and odorosides [18,19]. As expected, these major components are found in our combined extract. While extracts of Nerium oleander and Boswellia sacra demonstrated negligible or no significant inhibitory activity against respiratory syncytial virus (RSV) when tested at non-cytotoxic concentrations, and independently, the results changed significantly when the extracts were prepared in combination.

### 3.2. Antiviral Activity

Antiviral activity was investigated against a range of RNA and DNA viruses, including several important human pathogens. Among these natural products, NOBS7(1) was found to have interesting broad-spectrum antiviral activity; however, it is endowed with moderate cytotoxicity. NOBS7(1) showed an EC_50_ range of 0.1–1.3 μg/mL against RSV, herpesvirus simplex type 1, and coxsackie virus 5 (Table 1). 

**Figure 1:**
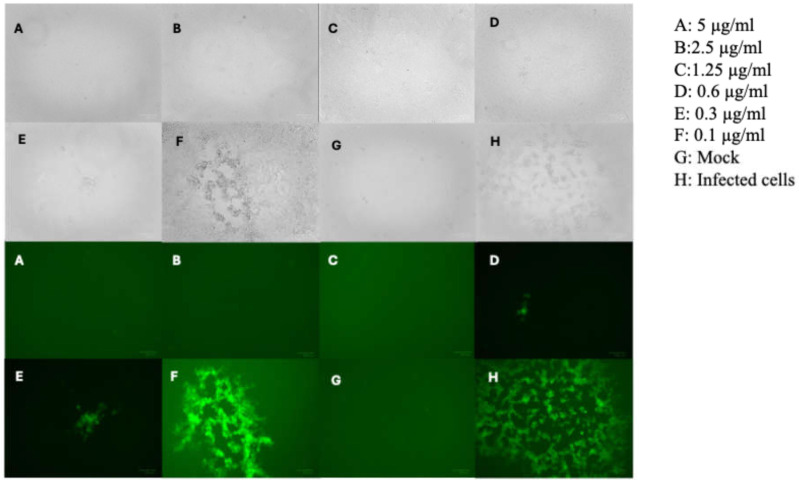
Inhibition of HSV-1 EGFP infection. Effect of NOBS7(1) inhibitor bright-field (above) and fluorescent images (below) at 5 µg/mL (**A**), 2.5 µg/mL (**B**), 1.25 µg/mL (**C**), 0.6 µg/mL (**D**), 0.3 µg/mL (**E**), and 0.1 µg/mL (**F**), and Mock (**G**) and infected cells (**H**) on the Vero-76 HSV-1 EGFP-infected monolayers. Pictures of cell morphology (up) were taken at 72 h post-infection using ZOE fluorescent cell imager (Bio-Rad) (bar size = 100 μm, magnification, 20×).

Interestingly, NOBS7(1) was not only endowed with anti-RSV activity (EC_50_ = 0.1 µM, SI = 90) but also promising anti-HSV-1 activity, resulting in the ability to protect Vero-76 monolayer from HSV-1 infection (Table 1, EC_50_ = 0.45 µM, SI = 20), as shown in Figure 1B. In contrast, NOBS8(2) and NOBS8(3) were selectively active against RSV (EC_50_s 1.5 and 1.8 μg/mL, respectively) with comparable cytotoxicity. Because NOBS7(1) exhibited a remarkable selectivity index against the respiratory virus RSV (SI = 90), we further investigated its potential mechanism of action against RSV. In parallel, NOBS8(3), less cytotoxic than NOBS8(2), and references were employed in our assay. The results of single *Boswellia sacra* extracts (BS7 and BS8) against representative viruses are shown in Table S1 in the Supplementary Section. No antiviral activity was detected against the broad spectrum of viruses or against RSV when tested alone.

Our research group has previously studied [10] *Nerium oleander* aqueous extracts (NO1, hot; NO2, cold) against several important human viral pathogens and found that when tested individually, none of them exhibited antiviral activity against RSV or HSV-1. Therefore, the antiviral results of the combined products against RSV and HSV-1 are a novel finding. 

Cytotoxicity was also analyzed using A549 cells, which are a standard model for studying respiratory virus infections, including RSV, influenza, and SARS-CoV-2, and Caco-2 cells, which are an in vitro epithelial cell model used for drug permeability assays in drug screening and compound toxicity testing. The results obtained are consistent with those obtained against Vero cells and are presented in Supplementary Table S3.

#### 3.2.1. NOBS7(1) and NOBS8(3) Effect on RSV Penetration into Pretreated Host Cells

To establish whether NOBS7(1) and NOBS8(3) were able to protect cells from RSV infection, a pre-attachment assay was then performed by incubating Vero-76 cell monolayers with the same concentration of NOBS7(1) and NOBS8(3) employed in the antiviral assay (5 and 10 μg/mL, respectively). Dextran sulphate was used as a reference compound. The eventual unbound drug was removed, cells were infected with RSV, and the inhibition of virus-induced CPE was recorded using an inverted light microscope after 5 days. Under these experimental conditions, NOBS7(1) and NOBS8(3) failed to inhibit RSV infection at the analyzed time point.

#### 3.2.2. Kinetics of RSV Adsorption in the Presence of NOBS7(1) and NOBS8(3) 

**Figure 2:**
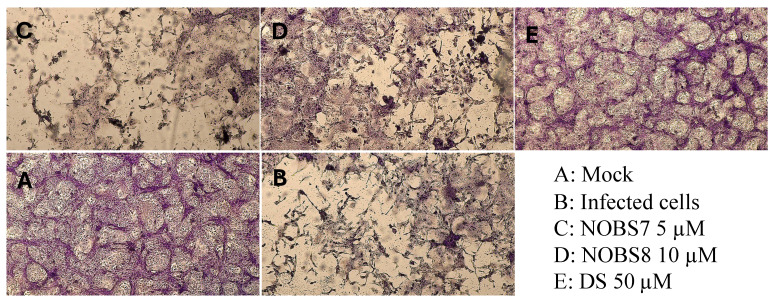
Kinetics of RSV adsorption in the presence of NOBS7(1) and NOBS8(3). Vero-76 cells were pre-adsorbed for 1 h at 4 °C with RSV at an m.o.i. of 0.1 in the presence of NOBS7(1) and NOBS8(3) (5 and 10 µg/mL). Dextran sulphate (DS) (50 µM) was employed as a reference. Medium containing unadsorbed virus was then removed, and cells were washed twice with PBS and overlayed with the medium. After 5 days of incubation, monolayers were stained with Crystal violet, and representative pictures of cell morphology were taken using the TiEseLab BDS 600 Inverted Microscope, GT CAM Model (4× magnification).

The treatment at low temperatures warrants the binding of viruses to the cell surface receptors but avoids the internalization of viral particles into the host cells. Accordingly, Vero-76 cells were incubated with RSV (m.o.i. = 0.1) and compounds NOBS7(1) and NOBS 8(3) for 2 h at 4 °C using fixed compound concentrations. The treatment with both NOBS7(1) and NOBS8(3) resulted in no detectable inhibition of virus-induced CPE in comparison to the untreated infected control (Figure 2). 

#### 3.2.3. Virucidal Activity of Compounds NOBS7(1) and NOBS8(3)

**Figure 3:**
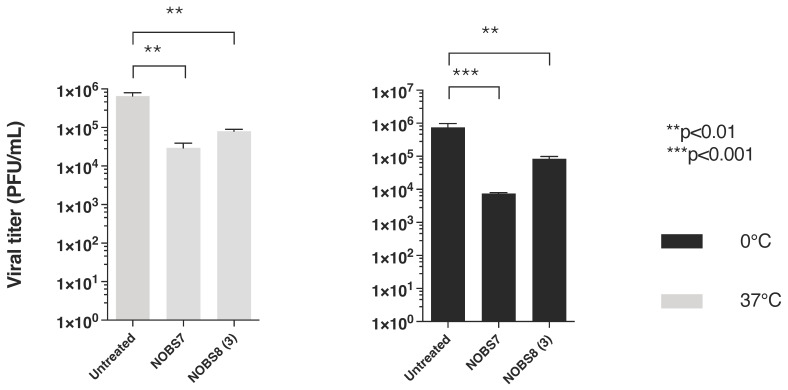
Virucidal activity of compounds NOBS7(1) and NOBS8(3) RSV A2 was pretreated for 2 h at either 0 °C or 37 °C with 5 and 10 µg/mL of NOBS7(1) and NOBS8(3), respectively. The mixture was then titrated on Vero-76 cells at high dilutions at which the concentration of compounds was not active. The titers are expressed as TCID_50_/mL. An ordinary one-way ANOVA was performed between the indicated groups. ** *p* < 0.01, *** *p* < 0.001.

To analyze the possibility that combined extracts NOBS7(1) and NOBS8(3) act directly on the virus particle, leading to infectivity inactivation, a virucidal assay against RSV virions was carried out. The potential virucidal effect of the two combined products was evaluated at a concentration of 5 and 10 μg/mL at either 0 °C or 37 °C. Significant differences between the titers of RSV treated at the two different temperatures are detected, as shown in Figure 3. The NOBS7(1) and NOBS8(3) concentrations were ten times higher than the compounds’ antiviral EC_50_, indicating that the inhibitory effect detected by the antiviral assay (0.1 and 1.5 μg/mL, respectively, as shown in Table 1) could be due to direct virion inactivation. 

**Figure 4:**
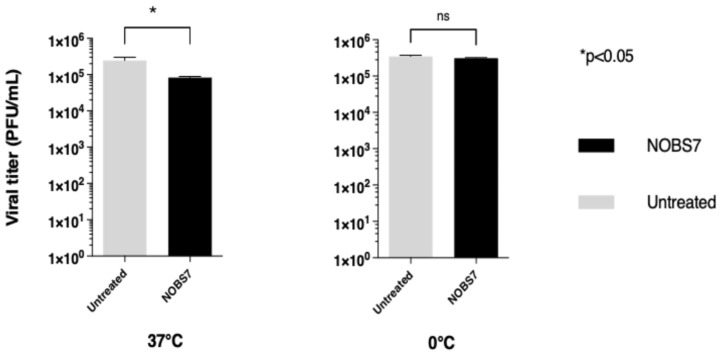
Virucidal activity of compound NOBS7(1). HSV-1 EGFP was pretreated for 2 h at either 0 °C or 37 °C with 5 µg/mL of compound. The mixture was then titrated on Vero-76 cells at high dilutions at which the concentration of compounds was not active. The titers are expressed as PFU/mL. An ordinary one-way ANOVA was performed between the indicated groups. * *p* < 0.05, ns = non-significant.

To validate the hypothesized mechanism of anti-RSV action of this combination of extracts, NOBS7(1) was selected to confirm the possible virucidal activity against HSV-1. As shown in Figure 4, HSV-1 was treated with 5 µg/mL NOBS7(1) for 2 h at 0 °C and 37 °C. The extract combination resulted in virucidal activity against HSV-1 when the virus was treated at 37 °C. No relevant reduction in plaque number was recorded when HSV-1 was treated with 5 µg/mL NOBS7(1) at 0 °C. The potential virucidal activity of NOBS7(1) was not validated against CVB5 because the EC_50_ was near the cytotoxicity (SI = 7). 

## 4. Discussion 

Natural extracts are a huge and sophisticated source of chemical library, offering a rich source of bioactive compounds for the development of novel antiviral drugs. Due to their exclusive molecular diversity, these extracts can inhibit viral infections through multiple mechanisms, such as blocking viral entry, disrupting genome replication, or modulating the host’s immune response. Notably, many natural compounds act as direct virucidal agents, meaning they can physically interact with and inactivate the virion itself. They neutralize the pathogen’s infectivity by disrupting the viral envelope or denaturing essential surface proteins. In the face of challenges such as increasing drug resistance and the emergence of novel pathogens, natural products remain crucial for global health. 

In this study, we evaluated the antiviral activity of natural compound mixtures NOBS7(1), NOBS8(2), and NOBS8(3) derived from *Nerium oleander* and *Boswellia sacra*, against a panel of human viruses. Although individual antiviral activities of *Boswellia sacra* (sacred frankincense) and *Nerium oleander* extracts have been extensively documented, research into their combined antiviral effects remains limited. As a primary step, we characterized the safety profile and cytotoxic potential of the combined products NOBS7(1), NOBS8(2), and NOBS8(3) in cell-based assays against uninfected Vero-76 cells (Table 1).

The cytotoxicity spectrum was also extended to A549 cells (Supplementary file, Table S3), a standard model in drug discovery research for studying replication of respiratory viruses. Comparable and mild cytotoxicity was observed in this cell line. The above assays suggest that antiviral assays could be conducted using low concentrations of all mixtures in order to observe the true antiviral effects independently of any adverse effects on the cells. Meanwhile, the compound mixtures were evaluated against a broad spectrum of important human pathogens. Antiviral activities were evaluated against RNA and DNA viruses. Although NOBS8(2) and NOBS8(3) showed moderate and selective activities on respiratory syncytial virus (RSV) or no significant antiviral effects under the tested conditions, NOBS7(1) demonstrated marked antiviral activity against RSV (Table 1). NOBS7(1) also exhibited moderate efficacy against the herpes simplex virus 1 (HSV-1), as demonstrated by its ability to protect Vero-76 monolayers against HSV-1 infection, as illustrated in Figure 1. Although research by Badria et al. (2003) [20] proved that a BA mixture from frankincense achieved a 100% reduction in viral plaque formation, to our knowledge, earlier literature has not reported the anti-RSV activity of *Nerium oleander* or Boswellia species extracts. To better understand the potential mode of action of NOBS7(1) and NOBS8(3), selected as endowed with better SI (15) than NOBS8(2), the Vero 76 cells were treated with active mixtures before being infected with RSV. The prophylactic treatment of Vero 76 cells with the active formulations did not reduce RSV infection levels. This lack of inhibitory activity suggests that the mixtures do not exert their effects through cellular protection or receptor blockade before viral entry. We also found that NOBS7(1) and NOBS8(3) were not able to reduce the RSV-induced CPE in the adsorption assay. When tested in a virucidal activity assay, NOBS7(1) and NOBS8(3) were found to successfully neutralize RSV infectivity. In particular, the virucidal assays demonstrated that NOBS7(1) and NOBS8(3) abrogated RSV infectivity at both 0 °C and 37 °C. These results suggest that the observed inactivation is temperature-independent within the tested conditions. 

To validate the proposed mode of action on RSV infectivity, we analyzed the virucidal activity of NOBS7(1) against HSV-1 virions. This compound mixture was less able to significantly reduce HSV-1 infectivity, and only at 37 °C. The weak but exclusive virucidal activity of our combined extract, NOBS7(1), against HSV-1 at 37 °C but not at 0 °C could be most likely explained by the critical requirement for membrane fluidity to enable extract penetration, glycoprotein interactions, and membrane disruption. At physiological temperature, the fluid state of the lipid bilayer enables these virucidal mechanisms. In contrast, at cold temperatures, the rigid, crystalline-like membrane organization protects the virus from extract-mediated inactivation [21]. The susceptibility of enveloped viruses to membrane-active virucidal agents is temperature-dependent, with maximum activity typically observed at physiological temperatures where membrane fluidity is optimal for agent penetration and viral envelope disruption [22]. Goswami et al. [6] previously described the anti-HSV-1 activity of *Boswellia serrata* oleo-gum resin and β-boswellic acid, which they found to be able to inhibit HSV-1 infection in vitro by modulating NF-κB and p38 MAPK signaling. Several studies [6,23,24,25,26,27] support the use of Boswellia in inhibiting the enzyme 5-lipoxygenase (5-LOX), thereby reducing the production of leukotrienes and alleviating inflammation associated with asthma and chronic bronchitis. Beyond the Boswellia established role in mitigating respiratory inflammation and asthma, the NOBS7(1) mixture demonstrated direct virucidal effects against human respiratory syncytial virus, which is a novelty and remains largely unexplored in existing literature. The observed activity in the combined product suggests that the simultaneous combination of both extracts creates a unique or synergistic chemical complex. This implies that the bioactivity is not simply the sum of its parts, but rather the result of a multifaceted molecular environment in which diverse classes of compounds (such as glycosides, triterpenes, and polysaccharides) work together to inhibit RSV infectivity. Other authors recently described [28,29] the anti-respiratory syncytial virus and anti-herpes simplex virus activity of polysaccharides from natural origin. The mode of action of these products involved a virucidal effect, the inhibition of viral entry, and additional subsequent effects. We are aware that our results are preliminary and that the mechanism of action we have identified requires further clarification. We could hypothesize that the NOBS extract does not act as a non-specific virucidal agent or ‘detergent’ (which would kill all enveloped viruses). Instead, it may act as a selective virucidal agent, exploiting the thermodynamic instability of the RSV fusion machinery and or its unique envelope composition.

## 5. Conclusions

The pharmaceutical industry has traditionally looked to natural products as a blueprint for drug design. This trend has accelerated in recent years, establishing natural products as a key source of modern therapeutic interventions. In the present study, we analyzed the antiviral effects of different *Boswellia sacra* and *Nerium oleander* combined extracts against a broad spectrum of viruses. The NOBS7(1) mixture was found to be highly active, demonstrating interesting virucidal effects against the human respiratory syncytial virus (hRSV). Further investigations are therefore warranted to fully elucidate the underlying molecular mechanisms and explore the potential for synergistic combinations of these natural constituents.

## Figures and Tables

**Table 1:** Cytotoxicity and broad-spectrum antiviral activity of natural extracts against the ssRNA+, enterovirus A71 (EVA71) and coxsackie virus B5 (CVB5), the ssRNA-, respiratory syncytial virus (RSV), measles virus (Measles), dsDNA vaccinia virus (VV), and herpes virus (HSV-1).

Compound	^a^ Vero-76	^b^ EVA71	^b^ RSV	^b^ Measles	^c^ HSV-1	^c^ VV	^c^ CVB5
	CC_50_ [μg/mL]	EC_50_ [μg/mL]					
NOBS7 (1)	9	>9	0.1 ± 0.05 (90)	>9	0.45 ± 0.15 (20)	>9	1.3 ± 0.6 (7)
NOBS8 (2)	8	>8	1.8 (4)	>8	>8	>8	>8
NOBS8 (3)	23	>23	1.5 (15)	>23	>23	>23	>23
**Reference Compounds**							
6-Azauridine	12 ± 2		1.2 ± 0.4	3.8			
NM 107	>100	1.4 ± 0.6					
M5255 (Mycophenolic acid)	80					2 ± 0.5	
Acyclovir					3 ± 0.5		
Pleconaril	>100						0.005 ± 0.001

Data represent mean values for three independent determinations. If not indicated, variation among samples was less than 15%. ^a^ Compound concentration (μg/mL) required to reduce the viability of mock-infected Vero-76 cells, as determined by the MTT method. ^b^ Compound concentration (μg/mL) required to achieve 50% protection of VERO-76 monolayers from the EVA71, determined by the MTT method, measles and RSV-induced cytopathogenicity, as determined by the Crystal violet uptake method. ^c^ Compound concentration (μg/mL) required to reduce the plaque number of VV (vaccinia virus), CVB5, and HSV-1, by 50% in Vero-76 monolayers. Reference concentrations are expressed in μM. (SI) Selectivity Index.

## Data Availability

The data that support the findings of this study are available from the corresponding authors, G.S. and L.R., upon reasonable request.

## Supplementary Materials

The following supporting information can be downloaded at: https://www.mdpi.com/article/10.3390/pathogens15030260/s1, Table S1: cytotoxicity and antiviral activity of *BS*, Table S2: chemical composition of compound mixtures, Table S3: cytotoxicity against A549 and Caco-2 cells.
