# Supplementary material for: New Insights into the Combined Antiviral Effect of Extracts from Nerium oleander and Boswellia sacra Against Respiratory Syncytial Virus: A Preliminary Report
Source: Pathogens. 2026 Mar 1;15(3):260. doi: 10.3390/pathogens15030260 (PMC13028964; doi:10.3390/pathogens15030260)
Supplement: Supplementary file 1 [file pathogens-15-00260-s001.zip › pathogens-4150701-supplementary.pdf]

Supporting Information

New insights into the combined antiviral effect of extracts from *Nerium oleander* and *Boswellia sacra* against Respiratory Syncytial virus: a preliminary report.

Rebecca Piras[1*], Luca Virdis[1*], Valeria Manca[1], Marta Cogoni[1], Vanessa Palmas[1], Matthew G. Donadu[2], Aldo Manzin[1], Giuseppina Sanna[1§], Luay Rashan[3§].

[1] Department of Biomedical Sciences, Microbiology and Virology Unit, University of Cagliari, Cittadella Universitaria, Sp. 8, 09042 Monserrato (CA), Italy,

[2] Hospital Pharmacy, Giovanni Paolo II Hospital, ASL Gallura, 07026 Olbia, Italy

[3] Dhofar University, Research Center, Dhofar University, Salalah, Oman

[*] Equal contribution

[§] Senior authorship and Correspondence: Emails: g.sanna@unica.it; lrashan@du.edu.om

Contents:

Table S1: Cytotoxicity and antiviral activity of *BS extracts*

Table S2: Chemical composition of compound mixtures

Table S3: Cytotoxicity against A549 and Caco-2 cells

Table S1: Cytotoxicity and antiviral activity of *BS extracts* and reference compounds against representatives of ssRNA+ (HIV-1, YFV, EVA71), ssRNA- (RSV), DNA (VV) viruses.

Compounds	MT-4	HIV-1	BHK-21	YFV	Vero-76	RSV	VV	EVA71
	[a]CC_{50}	[b]EC_{50}	[c]CC_{50}	[d]EC_{50}	[e]CC_{50}	[f]EC_{50}	[g]EC_{50}	[h]EC_{50}
BS2 100% Ethanol ext	22.5	>22.5	9	>9	21 (4dpi); 9	>9	>21	>21
BS3 50% Ethanol ext	27	>27	11	>11	12.5	>12.5	>12.5	>12.5
BS4 100% Methanol ext	15	>15	9	>9	20 (4dpi); 7.5	>7.5	>20	>20
BS5 Aqueous hot ext	>100	>100	>100	>100	>100	>100	>100	>100
BS6 Cold aqueous ext	>100	>100	>100	>100	>100	>100	>100	>100
BS7 Gum after hydrodistillation	8.5	>8.5	38	>38	23.8	>23.8	23.8	>23.8
BS8 Sugar fraction	78	>78	>100	>100	94	>94	>94	>94
References								
Efavirenz*	37	0.001						
2'-C-Me-Guo*			>100	1				1.4
6-Aza-Uridine*					14	2.2		
M5255					80		2	

[a]Compound concentration (μg/ml) required to reduce by 50% the proliferation of mock-infected MT-4 cells, as determined by the MTT method at day 4 following treatment.

[b]Compound concentration(μg/ml) required to achieve 50% protection of MT-4 cells from HIV-1-induced cytopathogenicity, as determined by the MTT method at day 4 p.i.

[c] Compound concentration(μg/ml) required to reduce by 50% the viability of mock-infected BHK cells, as determined by the MTT method at day 3 following treatment.

[d]Compound concentration(μg/ml) required to achieve 50% protection of BHK cells from YFV[(d)]-induced cytopathogenicity, as determined by the MTT method at day 3 p.i.

[e]Compound concentration(μg/ml) required to reduce by 50% the viability of mock-infected Vero-76 cells, as determined by the MTT method at day 5 following treatment

[f]Compound concentration(μg/ml) required to reduce by 50% the plaque number of RSV in Vero-76 cells at day 5 p.i.

[g] Compound concentration(μg/ml) required to reduce by 50% the plaque number of VV in Vero-76 cells at day 3 p.i.

[h]Compound concentration(μg/ml) required to achieve 50% protection of Vero-76 cells from EVA71-induced cytopathogenicity, as determined by the MTT method at day 4 p.i.

References concentrations are expressed in μM. (SI) Selectivity IndexData represent mean values for three independent determinations. If not indicated, variation among samples was less than 15%.

Table S2: Chemical composition of the combined extracts NOBS7(1) and NOBS8

NOBS8											
[a]GC-MS				[b]MS/MS							
**N.**	**Component**	**Cas**	**Content (%)**	**N.**	**Compound**	**Rt (min)**	**Formular**	**Calc. MW**	**m/z**	**Mode**	**Delta(ppm)**
1	Octamethylcyclotetrasiloxane	556-67-2	0.26	1	[6]-Gingerol	8.663	$C_{17}H_{26}O_4$	294.1841	293.1768	[M-H]-1	3.25
2	Decamethylcyclopentasiloxane	541-02-6	3.58	2	3α-Hydroxyglycyrrhetinate	11.255	$C_{30}H_{46}O_4$	470.3409	469.3337	[M-H]-1	2.8
3	Dodecamethylcyclohexasiloxane	540-97-6	11.41	3	Hederagenin	11.965	$C_{30}H_{48}O_4$	472.3567	471.3494	[M-H]-1	2.99
4	trans-Sobrerol[4-(1-hydroxy-1-methylethy-2-methyl-2-cyclohexen-1- ol]	997077-48-2	2.42	4	Ursolic acid	14.234	$C_{30}H_{48}O_3$	456.3611	455.3538	[M-H]-1	1.65

5	Tetradecamethylcycloheptasiloxane	107-50-6	5.92
6	6-Aza-5,7,12,14-tetrathiapentacene	997730-73-6	1.47
7	Octadecamethylcyclononasiloxane	556-71-8	0.90
8	Methyl 14-methylpentadecanoate	5129-60-2	1.30
9	β-Amyrin	559-70-6	1.20
10	2-(1-Methyl-1H-indol-3-yl)-3-[(trimethylsilyl)ethynyl]quinoxaline	997731-85-2	1.50
11	Hexadecanamide	629-54-9	3.01

5	Linolenic acid	10.534	$C_{18}H_{30}O_2$	278.2248	296.2586	$[M+NH_4]+1$	0.6
6	α-Curcumene	11.229	$C_{15}H_{22}$	202.1721	203.1794	[M+H]+1	-0.37
7	Chavicol	9.285	$C_9H_{10}O$	134.0732	135.0805	[M+H]+1	0.59
8	Cucurbitacin H	7.867	$C_{30}H_{46}O_8$	534.3197	535.327	[M+H]+1	0.78
9	Glycyrrhetinic Acid	11.926	$C_{30}H_{46}O_4$	470.3399	471.3472	[M+H]+1	0.66

12	(Z)-9-Octadecenamide	301-02-0	23.27
13	Octadecanamide	124-26-5	6.03
14	Silicone oil	000-00-0	0.67
15	(Z)-Docos-9-enenitrile	997625-36-8	1.76
16	Tetracosamethylcyclododecasiloxane	18919-94-352	0.47
17	(Z)-13-Docosenamide	112-84-5	4.23
18	24-Norursa-3,9(11),12-triene	930591-91-6	1.30
19	24-Noroleana-3,12-diene	201358-24-9	3.97
20	Vasicionolone	997265-67-7	9.40

21	24-Norursa-3,12-dien-11-one	930591-92-7	9.99

[a]Compounds identified in NOBS8 extracts by GC–MS analysis; relative abundance is expressed as percentage of total ion current (%).

[b]Compounds identified by MS/MS analysis based on retention time (Rt), molecular formula, calculated molecular weight (MW), experimental *m*/*z* values, ionization mode, and mass accuracy (Δ, ppm).

NOBS7(1)											
[a]GC-MS				[b]MS/MS							
N.	Component	Cas	Content (%)	N.	Compound	Rt (min)	Formular	Calc. MW	m/z	Mode	Delta(ppm)
1	4-Vinylphenol	2628-17-3	1.45%	1	[6]-Gingerol	8.658	$C_{17}H_{26}O_4$	294.1841	293.1768	[M-H]-1	3.35
2	Dodecamethylcyclohexasiloxane	540-97-6	2.50%	2	3α-Hydroxyglycyrrhetinate	11.396	$C_{30}H_{46}O_4$	470.3411	469.3338	[M-H]-1	3.06
3	3-Cyclohexene-1-methanol, 5-hydroxy-α,α,4-trimethyl-	498-71-5	0.52%	3	Caffeic Acid	4.33	$C_9H_8O_4$	180.0428	179.0356	[M-H]-1	3.17
4	Heptanal	111-71-7	1.31%	4	Canangalia C	7.286	$C_{17}H_{28}O_6$	328.19	327.1827	[M-H]-1	4.16
5	Methyl 3,5-dichlorobenzoate	2905-67-1	0.23%	5	Catechol	3.055	$C_6H_6O_2$	110.0372	109.0299	[M-H]-1	3.95
6	Glutaric acid, di((2-methylcyclohex-1-	997713-50-8	0.29%	6	Geranylacetate	7.7	$C_{12}H_{20}O_2$	196.1469	195.1397	[M-H]-1	3.03

	enyl)methyl) ester										
7	2-Methoxy-4-propylPhenol	2785-87-7	0.88	7	Salicylic acid	5.987	$C_7H_6O_3$	138.0321	137.0248	[M-H]-1	2.74
8	Tetradecamethylcycloheptasiloxane	107-50-6	3.02	8	Ursolic acid	13.545	$C_{30}H_{48}O_3$	456.3613	455.3541	[M-H]-1	2.18
9	3-Methyl-7-phenyl-pyrazolo[1,5-d]tetrazole	59772-86-0	0.97	9	(+)-Echinoisoflavanone	6.558	$C_{22}H_{24}O_7$	400.1526	401.1599	[M+H]+1	0.99
10	Homovanillyl alcohol	2380-78-1	0.72	10	Linolenic acid	10.526	$C_{18}H_{30}O_2$	278.2247	296.2585	[M+NH$_4$]+1	0.4
11	1-Hydroxy-9-oxabicyclo[3.3.1]nonane	999041-00-8	0.40	11	3-Oxoglycyrrhetinate	10.769	$C_{30}H_{44}O_4$	468.3244	469.3316	[M+H]+1	0.85
12	4-ethenyl-2,6-dimethoxy-phenol	997146-04-5	0.33	12	α-Curcumene	10.633	$C_{15}H_{22}$	202.1721	203.1794	[M+H]+1	-0.18
13	Megastigmatrienone	38818-55-2	0.40	13	α-Cyperone	10.667	$C_{15}H_{22}O$	218.167	219.1743	[M+H]+1	-0.33

14	2,5-Di-O-acetyl-3,4,6-tri-O-methyl-D-mannonitrile	58720-08-4	0.18
15	2-[[4-(1,1-Dimethylethyl)phenoxy]methyl]oxirane	3101-60-8	0.27
16	4-[(1E)-1,3-Butadienyl]-3,5,5-trimethyl-2-cyclohexen-1-one	38818-55-2	0.29
17	Hexadecamethylcyclooctasiloxane	556-68-3	2.93
18	2-Methyl-2-ethyldioxolane	126-39-6 35	55.32
19	Syringylacetone	19037-58-2	0.21

14	α-Santonin	8.174	$C_{15}H_{18}O_3$	246.1256	247.1329	[M+H]+1	0.07
15	β-Ionone	6.673	$C_{13}H_{20}O$	192.1515	193.1588	[M+H]+1	0.56
16	Digitoxigenin	8.268	$C_{23}H_{34}O_4$	374.2459	375.2532	[M+H]+1	0.54
17	Glycyrrhetinic Acid	11.927	$C_{30}H_{46}O_4$	470.34	471.3472	[M+H]+1	0.73

20	2-Methyldiphenylsulfone	7018-84-0	0.63
21	Octadecamethylcyclononasiloxane	556-71-8	2.55
22	4,6-bis(1,1-dimethylethyl)-3-hydroxy 2H-pyran-2-one	65077-06-7	0.24
23	Phthalic acid, 2-methylbutyl pentyl ester	997580-96-7	0.24
24	Phthalic acid, isobutyl 2-(4-nitrophenoxy)ethyl ester	997806-66-7	1.53
25	Hexadecanoic acid, methyl ester	112-39-0	0.22
26	7,9-Di-tert-butyl-1-oxaspiro(4,5)deca-6,9-	82304-66-3	0.14

	diene-2,8-dione		
27	Eicosamethyl cyclodecasiloxane	18772-36-6	2.45
28	3-(4-N,N-Dimethylaminophenyl)propenoic acid, 2-(diethoxyphosphinyl)-, ethyl ester	66564-08-7	2.02
29	Hexadecanamide	629-54-9	0.71
30	Silicone oil	0	28.00
31	Elaidamide	301-02-0	2.68
32	Octadecanamide	124-26-5	0.57
33	(Z)-13-Docosenamide	112-84-5	2.92
34	Pentalen-13-al	997268-43-6	16.00

[a]Compounds identified in NOBS7(1) extracts by GC–MS analysis; relative abundance is expressed as percentage of total ion current (%).

[b]Compounds identified by MS/MS analysis based on retention time (Rt), molecular formula, calculated molecular weight (MW), experimental *m/z* values, ionization mode, and mass accuracy (Δ, ppm).

Table S3: Antiproliferative activity of *Boswellia sacra* extracts (BS7, BS8) before and after combination (NOBS7(1) and NOBS8(2&3) with *Nerium oleander* cold extract (NO11)

**Compound**	**[b]A549**	**[c]Caco-2**
	**[a]CC_{50} [µg/ml]**	
**BS7**	7±0.2	12±3
**BS8**	18.2±0.02	20±2
**NO11**	5.1±0.05	7±05
**NOBS7(1)**	5±0.1	6±0.5
**NOBS8(2)**	16.5±0.05	18±2
**NOBS8(3)**	17.2±0.02	16±0.8

[a]Compound concentration required to reduce cell viability by 50%, as determined by the MTT method, under conditions allowing untreated controls to undergo at least three consecutive rounds of multiplication [b]Human pulmonary adenocarcinoma, [c]Human colorectal adenocarcinoma. Data represent mean values for three independent determinations (for Caco-2, two independent determinations). If not indicated, variation among samples was less than 15%.
